# Memory in Misfire: The Gut Microbiome-Trained Immunity Circuit in Inflammatory Bowel Diseases

**DOI:** 10.3390/ijms26199663

**Published:** 2025-10-03

**Authors:** Binbin Yang, Jiacheng Wu, Xiaohua Hou, Tao Bai, Shi Liu

**Affiliations:** Division of Gastroenterology, Union Hospital, Tongji Medical College, Huazhong University of Science and Technology, Wuhan 430062, China; m202476204@hust.edu.cn (B.Y.); m202376082@hust.edu.cn (J.W.); houxh@hust.edu.cn (X.H.)

**Keywords:** innate immune memory, trained immunity, inflammatory bowel diseases, gut microbiome

## Abstract

Inflammatory bowel disease (IBD) demonstrates chronic relapsing inflammation extending beyond adaptive immunity dysfunction. “Trained immunity”—the reprogramming of innate immune memory in myeloid cells and hematopoietic progenitors—maintains intestinal inflammation; however, the mechanism by which gut microbiome orchestration determines protective versus pathological outcomes remains unclear. Microbial metabolites demonstrate context-dependent dual effects along the gut–bone marrow axis. Short-chain fatty acids typically induce tolerogenic immune memory, whereas metabolites like succinate and polyamines exhibit dual roles: promoting inflammation in certain contexts while enhancing barrier integrity in others, influenced by cell-specific receptors and microenvironmental factors. Interventions include precision probiotics and postbiotics delivering specific metabolites, fecal microbiota transplantation addressing dysbiotic trained immunity, targeted metabolite supplementation, and pharmacologic reprogramming of pathological myeloid training states. Patient stratification based on microbiome composition and host genetics enhances therapeutic precision. Future research requires integration of non-coding RNAs regulating trained immunity, microbiome–immune–neuronal axis interactions, and host genetic variants modulating microbiome–immunity crosstalk. Priorities include developing companion diagnostics, establishing regulatory frameworks for microbiome therapeutics, and defining mechanistic switches for personalized interventions.

## 1. Introduction

Inflammatory bowel disease (IBD), encompassing Crohn’s disease (CD) and ulcerative colitis (UC), is a chronic relapsing inflammatory disorder affecting the gastrointestinal mucosa. Despite therapeutic advances, IBD remains highly prevalent, causes substantial morbidity, and increases colorectal cancer risk [1,2]. Recurrent abdominal pain, diarrhea, and rectal bleeding compromise quality of life and impose significant healthcare costs [3]. Current pharmacological treatments, including biologics, demonstrate inconsistent efficacy with marked inter-patient variability and limited long-term remission rates [4,5,6,7]. Precision therapeutic strategies are therefore essential to prevent disease progression and complications.

Innate immunity is central to IBD pathogenesis and persistence. Genetic variants in NOD2 and CARD9 disrupt innate signaling, triggering aberrant microbial pattern recognition that compromises barrier integrity and activates adaptive immunity [8,9]. Sustained innate cell activation drives chronic inflammation and fibrogenesis.

Critically, innate cells acquire non-specific “trained” memory through epigenetic and metabolic reprogramming, which in IBD’s inflammatory environment perpetuates pro-inflammatory cytokine production and fibrosis [8,10,11]. Following initial insult, monocytes, macrophages, and innate lymphoid cells exhibit heightened cytokine responses upon re-challenge [12]. While trained immunity normally provides pathogen protection, its dysregulation sustains pathological inflammation. Understanding the signals establishing and maintaining this memory is therefore crucial for explaining IBD chronicity.

The gut microbiome provides many such signals. Dysbiosis depletes beneficial taxa while enriching pathobionts, altering luminal concentrations of short-chain fatty acids, secondary bile acids, and tryptophan metabolites [13,14]. These molecules function as epigenetic cofactors and metabolic substrates that program trained immunity in innate cells [12]. Modulating microbial composition or supplementing key metabolites thus offers a practical approach to recalibrate pathogenic innate memory.

Here, we synthesize current understanding of the innate immunity–microbiota axis in IBD and highlight therapeutic strategies targeting trained immunity for precise, durable disease control.

## 2. Mechanism and Plasticity of Trained Immunity

Trained immunity refers to the non-specific immune memory acquired by innate immune cells following an initial stimulus such as infection, vaccination, or inflammatory factors, first proposed by Netea et al. in 2011 [15]. Unlike adaptive immunity, which relies on T and B cells, trained immunity involves functional reprogramming of monocytes, macrophages, NK cells, and innate lymphoid cells [16]. This process is primarily driven by epigenetic reprogramming, including histone modifications and DNA methylation, which persist even after the stimulus is removed, allowing cells to respond more rapidly and robustly to future exposures [17,18,19]. Key epigenetic changes include histone lactylation (H3K18la) and DNA methylation (e.g., H3K4me3), regulated by immune-initiating lncRNAs, which guide chromatin modifications at immune-related gene promoters [20,21,22]. These “imprints” enable innate cells to initiate stronger immune responses upon subsequent encounters with similar or different stimuli, conferring a memory-like feature with broad adaptability [10,16]. Besides, trained immunity differs from adaptive immunity in IBD pathogenesis (Table 1). These differences not only explain the rapid nonspecific recurrence and treatment resistance of IBD that traditional adaptive immune theories cannot account for but also provide new therapeutic targets and time windows. Furthermore, they suggest that therapies capable of simultaneously targeting both forms of immune memory may prove more effective.

**Figure 1:**
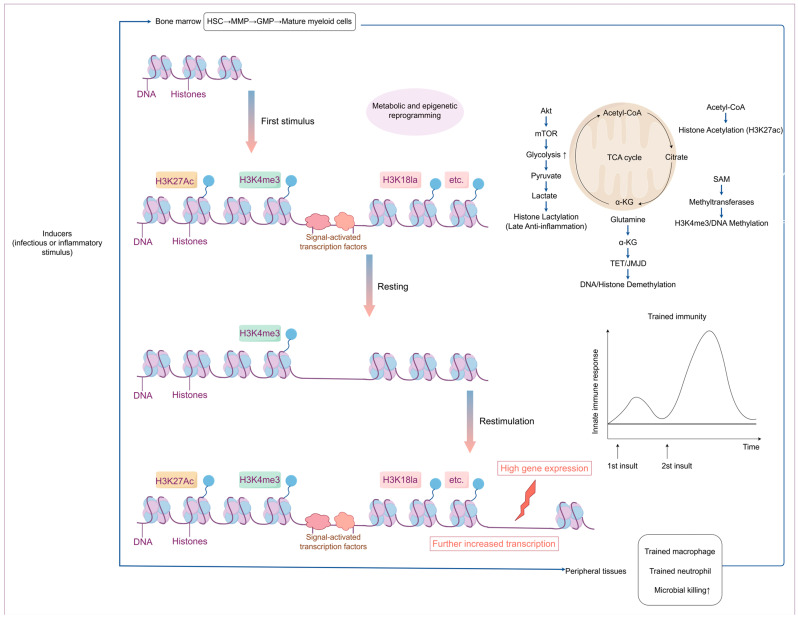
Overview of the epigenetic and metabolic mechanisms of trained immunity.

Metabolic reprogramming is one of the key drivers of trained immunity (Figure 1), which provides energy to immune cells and participates in epigenetic modification and signaling through the production of specific metabolic intermediates to maintain the trained state at the functional level. It was found that during trained immunity, innate immune cells undergo significant metabolic pathway switching, which is manifested by enhanced aerobic glycolysis (Warburg effect), redistribution of the tricarboxylic acid cycle (TCA) and oxidative phosphorylation, and other metabolic dynamics. This reprogramming results in increased glucose uptake, significantly elevated levels of glycolysis, and rapid conversion of pyruvate to lactate, which provides rapid cellular energy and promotes activation of pro-inflammatory signaling [39]. At the same time, some TCA cycle intermediates have signaling functions of their own [40]. Succinate is a key metabolic signaling molecule that activates pro-inflammatory pathways and enhances the expression of inflammatory factors through its receptor SUCNR1 [41]. Succinate accumulation is closely associated with developing an inflammatory phenotype in innate immune cells (e.g., macrophages) and is an essential metabolic marker of trained immunity [42].

Multiple infectious or inflammatory stimuli can trigger central trained immunity (TRIM) in the bone marrow. This mechanism encompasses metabolic and epigenetic reprogramming of hematopoietic stem and progenitor cells, including hematopoietic stem cells (HSCs), multipotent progenitors (MPPs), and granulocyte–macrophage progenitors (GMPs). This reprogramming leads to increased production of mature myeloid cells (neutrophils and monocytes) with heightened immune responsiveness to subsequent challenges encountered in the circulation or peripheral tissues. TRIM can also be directly induced in mature myeloid cells within the periphery (circulation or peripheral tissues and organs). Epigenetic marks—such as H3K4me3—can persist without the initial stimulus, facilitating more robust transcription of immune genes upon secondary stimulation and resulting in an amplified immune response [17].

Trained immunity is highly plastic. Established studies have shown that infection or vaccination induces durable epigenetic modifications in innate immune cells, which enhance their ability to respond to subsequent pathogenic or inflammatory stimuli. For example, trained immunity induced by the BCG vaccine (the primary vaccine against *Mycobacterium tuberculosis* worldwide) not only boosts immune protection against *Mycobacterium tuberculosis* but also offers cross-protection against heterologous infections [27,43,44]. This process might be achieved by inducing HIF1α and mTOR-mediated metabolic reprogramming of monocytes [45,46]. More importantly, trained immunity is not limited to peripheral innate immune cells, and its epigenetic reprogramming can also be established in immune progenitor cells in the bone marrow, explaining that some vaccines can maintain their protective effect against heterologous infections for years [19]. However, trained immunity, when excessive or dysregulated, can play a disease-promoting role in chronic inflammatory diseases. Following an initial infection or inflammatory challenge, trained immunity is epigenetically reprogrammed via bone marrow hematopoietic stem and progenitor cells (BM-HSPCs) to form long-term inflammatory memories in response to inflammatory signals, which amplify and delay chronic inflammatory pathologies [47]. Notably, trained immunity represents a double-edged sword. For example, in systemic sclerosis (SSc), BCG-trained macrophages exacerbate the fibrosis process. In contrast, low-dose LPS pre-trained macrophages attenuate the disease [48], which reveals that trained immunity can exhibit opposite functional effects under different stimulus conditions.

## 3. Microbiome Regulation of Trained Immunity

### 3.1. Exposure to Bacterial Flora Directly Regulates Trained Immunity

**Figure 2:**
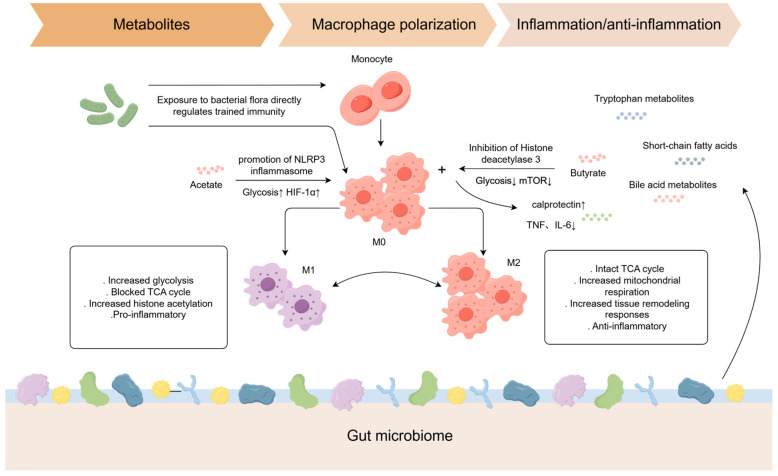
How microbes and their metabolites induce different types of trained immunity.

Commensal and pathogenic microbes, their components, vaccines, and microbial metabolites can all reprogram innate immune cells through epigenetic and metabolic mechanisms (Figure 2), producing a spectrum from trained immunity to tolerance. The specific outcome is shaped by the agent involved, the cell type, the context of the interaction, and the host tissue environment, which provides opportunities for both targeted mucosal protection and the maintenance of intestinal immune homeostasis (Table 2) [12,49,50,51,52,53,54,55,56,57,58,59,60,61,62]

Microbiota metabolites regulate macrophage polarization through metabolic reprogramming [63]. Butyrate, a short-chain fatty acid (SCFA), modulates the epigenetic landscape of intestinal macrophages by inhibiting histone deacetylase (HDAC), enhancing antimicrobial capacity (e.g., upregulating calprotectin expression) while suppressing pro-inflammatory cytokine release (e.g., TNF and IL-6), thereby promoting intestinal homeostasis [64,65]. Acetate enhances macrophage bactericidal activity via NLRP3 inflammasome activation and glycolysis-mediated HIF-1α induction [66].

### 3.2. Microbial Metabolite-Mediated Regulation of Trained Immunity

Microbial metabolites are pivotal modulators of trained immunity. The establishment and maintenance of trained immunity rely on the involvement of various metabolic pathways, including aerobic glycolysis, glutamine catabolism, cholesterol metabolism, and fatty acid synthesis. All these pathways are intimately connected to the metabolic activities of intestinal microbes. These microbial metabolites influence innate immune cells both directly (Figure 2), by altering their epigenetic landscape, and indirectly, through activation of immunoregulatory pathways in intestinal epithelial cells.

Recent years have witnessed substantial advances in understanding how specific microbial metabolites regulate trained immunity [63]. Notably, short-chain fatty acids (SCFAs) have been repeatedly shown to induce trained immunity via epigenetic and metabolic reprogramming of innate immune cells [12]. Beyond SCFAs, other metabolites such as lactic acid can serve as metabolic fuels for the tricarboxylic acid cycle and, through histone lactylation, increase chromatin accessibility, thus enhancing immune responsiveness to secondary stimuli [67]. The aromatic compound resorcinol, via AhR pathway activation, epigenetically reprograms hematopoietic stem and progenitor cells (HSPCs) to confer central trained immunity [32]. Furthermore, tryptophan-derived metabolites (e.g., p-cresol sulfate and indole-3-glyoxalate) amplify inflammatory responses in the CNS by activating AhR and promoting IL-17 production from γδ T cells and Th17 cells [68].

Other microbial products, including γ-aminobutyric acid (GABA), polyamines, and histamine, have also been implicated in immune regulation, partly through epigenetic modifications. Additionally, intermediates from the mevalonate pathway promote trained immunity maintenance by activating the IGF1-mTOR signaling axis and driving histone modification [69].

Notably, host–microbe interactions are bidirectional. For example, apolipoprotein L (APOL9a/b) secreted by intestinal epithelial cells can enhance the release of bacterial outer membrane vesicles (OMVs), thereby activating TLR2 signaling in dendritic cells and facilitating IFN-γ secretion and MHC-II upregulation, which together promote intraepithelial lymphocyte differentiation [70]. These findings underscore the intricate interplay between microbial metabolites, host cells, and immune function.

### 3.3. Host Genetics: A Key Modulator of the Microbiome–Immunity Axis

While the gut microbiome regulates trained immunity, this regulation is fundamentally shaped by host genetic susceptibility. IBD pathogenesis involves over 200 genetic risk loci, many encoding proteins essential for innate immunity, microbial recognition, and autophagy, including NOD2, CARD9, and ATG16L1 [71,72,73]. These genetic variants predispose to dysregulated host–microbe interactions. Loss-of-function NOD2 variants impair muramyl dipeptide sensing, causing defective Paneth cell antimicrobial function and subsequent dysbiosis characterized by increased Enterobacteriaceae abundance [74]. Similarly, CARD9 polymorphisms alter gut microbiome composition and anti-fungal immune responses [75,76]. Individual genetic profiles thus determine microbial composition and immune setpoints. This genetically driven dysbiosis provides persistent aberrant stimuli, priming innate immunity for maladaptive trained responses that contribute to IBD chronicity. Additionally, non-coding RNAs, particularly long non-coding RNAs (lncRNAs), increasingly influence trained immunity. LncRNAs regulate histone modifications (H3K4me3 and H3K27me3) and DNA methylation through chromatin modification complex binding, while modulating immune cell metabolic pathways—glycolysis and oxidative phosphorylation—by controlling metabolic enzyme expression and activity [77,78]. Studies demonstrate non-coding RNA’s roles in IBD pathogenesis; inhibiting lncRNA NEAT1 attenuates IBD inflammatory responses [79]. These findings suggest non-coding RNAs shape trained immunity in IBD, though this emerging field requires further definitive research.

## 4. Tolerogenic Trained Immunity and Bidirectional Regulation by Microbial Metabolites

Although trained immunity is typically linked to pro-inflammatory memory, recent studies demonstrate that specific commensal and probiotic bacteria can induce a tolerogenic form, characterised by dampened inflammatory responses and promotion of immune homeostasis. For instance, *Lactiplantibacillus plantarum* imprints tolerogenic memory in mononuclear phagocytes by downregulating ROS production and reprogramming metabolic pathways, thereby reducing TNF-α and IL-1β upon restimulation [50]. Similarly, *Akkermansia muciniphila* modulates macrophage training through mitochondrial and lysosomal signaling, enhancing immune tolerance and barrier protection [51]. These effects, which depend on viable bacteria and are influenced by environmental cues such as retinoic acid and mucosal oxygen, involve distinct epigenetic marks (e.g., H3K27me3 enrichment) and suppression of NF-κB signaling mechanistically [51].

Notably, the influence of microbial metabolites on intestinal inflammation via trained immunity is complex. Clinical and preclinical evidence suggests that the anti-inflammatory effects of certain short-chain fatty acids (SCFAs) are linked to trained immunity [80,81,82]. For example, the SCFA butyrate inhibits histone deacetylases, particularly HDAC3, in intestinal macrophages, thereby promoting an anti-inflammatory phenotype, enhancing epithelial barrier integrity, and limiting excessive type 2 immunity to support mucosal homeostasis [64,65,83,84]. In contrast, succinate exhibits context-dependent effects: chronic elevation in IBD can drive pro-inflammatory pathways through SUCNR1 signaling and Th17 activation, whereas controlled dietary supplementation activates the IL-4Rα–Hif-1α axis to induce anti-inflammatory M2 macrophages and promote tissue repair [31,85,86]. Polyamines demonstrate similar duality. In IBD, physiological levels of spermidine promote macrophage polarisation toward an anti-inflammatory M2 phenotype, a protection dependent on epithelial and myeloid PTPN2 [87], whereas dysregulated polyamine levels and metabolism are associated with persistent inflammation and tissue damage [88,89]. Given that both excessive and insufficient immune activation can exacerbate intestinal pathology, precise modulation of these metabolite levels may offer a promising approach to restoring immune balance in chronic inflammatory conditions such as IBD.

## 5. Critical Pathways of the Flora–Immune Memory Axis Driving IBD Chronicity

### 5.1. The Gut–Bone Marrow Axis

**Figure 3:**
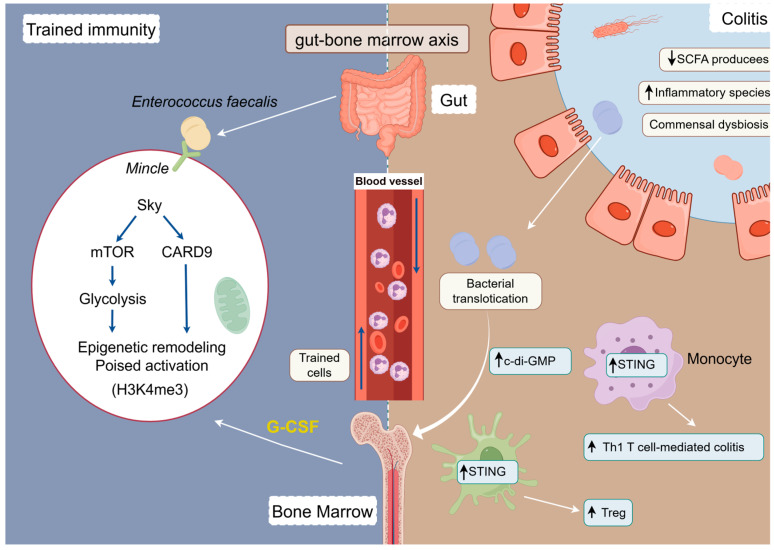
Modelling of the ‘gut–bone marrow axis’ tele-training immune pathway.

In recent years, the concept of the “gut–bone marrow axis” has been proposed, suggesting that signals from the gut flora may act remotely on the bone marrow hematopoietic system to induce trained immunity, which is involved in the maintenance and amplification of chronic inflammation in IBD [32,90]. In the DSS-induced mouse colitis model, Mincle-mediated trained immunity significantly enhances the body’s protective immune response to subsequent bacterial and viral infections (Figure 3). However, it may also exacerbate the pathologic course of the associated disease by enhancing the inflammatory response in the presence of intestinal barrier damage [36]. Upon intestinal barrier damage, ZO-1 degradation leads to translocation of enterococci to the bone marrow, triggering activation of bone marrow immune cells and transmission of immune memory [91]. Simultaneously, CX3CR1^+^ monocytes act as “sentinels” that capture LPS and transport it to the bone marrow niche, activating immune cells in the bone marrow and transmitting immune memory signals [91]. In contrast to the traditional “bone marrow → periphery” linear hematopoietic model, this “gut–bone marrow–periphery” pathway reveals the dynamic capacity of gut signals to shape the fate of bone marrow immune cells. Trained immunity is thus no longer merely an activation of the end-cell state, but a functional reprogramming of early hematopoietic progenitors. Their “memory” alterations can maintain a pro-inflammatory phenotype over time, providing a deeper driving mechanism for the chronicity of IBD. In another study, a constitutively activated mouse model of STING (N153s) revealed that intestinal dysbiosis induces K63 ubiquitination modification of STING through the bacterial metabolite c-di-GMP, leading to the accumulation of innate immune junction protein STING in myeloid cells (macrophage/monocyte), activation of the TBK1/IRF3/NF-κB pathway, driving the release of pro-inflammatory factors (e.g., TNF-α and CXCL10) and promoting T cell-dependent colitis [92].

The trans-barrier translocation of gut microbiota, particularly *Enterococcus fecalis*, induces a long-term state of trained immunity by activating the Mincle receptor on bone marrow myeloid progenitor cells [36]. And intestinal dysbiosis induces K63 ubiquitination modification of STING through the bacterial metabolite c-di-GMP [92].

### 5.2. Deficiency of Protective Metabolites and Pathological Training of Pro-Inflammatory Metabolites

Microbial metabolites not only exert remote effects but also directly modulate trained immunity within the intestinal microenvironment, thereby influencing IBD pathogenesis [85]. In IBD patients, the loss of SCFA-producing bacteria and tryptophan-metabolising bacteria leads to reduced levels of key metabolites such as butyrate and indole derivatives [63,93]. These metabolites are essential for the regulation of innate immune cell metabolic programming and epigenetic remodelling, both of which underpin trained immunity. For example, butyrate has been shown to inhibit histone deacetylase (HDAC) activity in monocytes and neutrophils, suppressing excessive inflammatory responses and modulating trained immunity [93]. Moreover, colonisation experiments in germ-free mice indicate that the gut microbiota from IBD patients can enhance glycolytic activity in bone marrow CD11b+ cells, further supporting the role of microbial-driven metabolic reprogramming in shaping trained immunity [94,95]. These findings collectively highlight how altered microbial composition and metabolite availability in IBD contribute to dysregulated trained immunity and the maintenance of chronic intestinal inflammation.

### 5.3. The Microbiome–Immune–Neuronal Axis

The microbiota–gut–brain axis has been demonstrated to play a pivotal role in the pathogenesis of IBD [96]. Indeed, the immune system is also involved, forming the microbiota–immune–neural axis—another complex bidirectional communication system [97]. Within this framework, microbial metabolites serve as key hubs for neuro-immune coupling, acting on sensory neurons (TRPV1/TRPA1, P2X3), G protein-coupled receptors (GPR41/43/109A), nuclear receptors/transcription factors (AhR, FXR/TGR5), and immune receptors (TLR2/4/5), collectively shaping intestinal inflammation, pain sensitization, and behavioral/cognitive alterations [98,99,100]. As previously described, microbial metabolites can regulate host inflammatory memory through epigenetic and metabolic reprogramming. Indeed, beyond influencing neuronal signaling and immune memory in IBD, microbial metabolites also affect extraintestinal manifestations of IBD. When intestinal barrier function is compromised, associated protective mechanisms fail. Circulating proinflammatory cytokines (e.g., IL-6 and TNF-α) can then cross the damaged blood–brain barrier (BBB), activating resident brain immune cells such as microglia and inducing neuroinflammation. This may provide a pathological explanation for the psychiatric and neurological symptoms observed in IBD patients [101]. Current animal studies have demonstrated the beneficial effects of targeting the microbiome–immune–neural axis on IBD remission [100,102].

### 5.4. Transgenerational Transmission

More notably, several studies in recent years have suggested that the synergistic effect of gut flora and epigenetic mechanisms may be involved in the transgenerational transmission of trained immunity [103,104]. It has been shown that in granulopoietic mice with established trained immunity, granulocyte–monocyte progenitors (GMPs) exhibit a strong type I interferon signaling signature that correlates with type I interferon-dependent epigenetic imprinting in the progeny’s neutrophils, ultimately leading to a significant enhancement of oxidative burst and phagocytosis in these cells in response to subsequent stimulation [105]. In addition, changes in maternal diet and gut flora can influence the training status of immune cells in the offspring through epigenetic mechanisms such as miRNAs in the sperm or egg, which in turn affects their health [106]. Moreover, IBD familial aggregation may partly arise from vertical transmission of colony–immune memory [107]. These findings emphasise the profound influence of maternal microecological and epigenetic status on the risk of IBD in the offspring and provide new ideas for early intervention of the disease.

## 6. Intervention Strategies Targeting the Flora–Immune Memory Axis

The intricate interactions between the intestinal microbiota and the immune system offer promising therapeutic avenues for IBD. The “diet–gut microbiota–innate immunity” theoretical framework provides systematic guidance for treating IBD. Below, we outline intervention strategies at the levels of primary prevention, secondary treatment, and tertiary (long-term) management, highlighting evidence-based approaches supported by recent literature.

### 6.1. Primary Prevention

Maintaining intestinal flora homeostasis through dietary interventions in healthy populations or at-risk individuals can help reduce the risk of developing IBD [108]. This goes beyond generic healthy eating to specific dietary components that foster intestinal flora homeostasis and beneficial immune training.

#### 6.1.1. Dietary Fibre and Whole Grains

A high intake of diverse dietary fibres and whole grains is associated with a lower risk of developing IBD [109]. These complex carbohydrates are indigestible by human enzymes but fermentable by gut bacteria, leading to the production of short-chain fatty acids (SCFAs) such as butyrate [110]. SCFAs play a vital role in maintaining gut barrier integrity, upregulating tight junction proteins, and modulating immune responses towards an anti-inflammatory profile. In a large prospective cohort study, individuals with the highest fibre intake (especially from cereals and whole grains) had a significantly reduced incidence of Crohn’s disease and ulcerative colitis [111]. This evidence reinforces dietary fibre as a cornerstone of IBD prevention. SCFA-mediated mechanisms further explain this benefit: butyrate serves as an energy source for colonic epithelial cells and promotes regulatory T-cell development, thereby enhancing mucosal tolerance.

#### 6.1.2. Prebiotics

Beyond general fibre, specific prebiotic compounds support beneficial gut microbes like *Bifidobacterium* and *Lactobacillus*. Fructans (e.g., inulin from onions, garlic, and bananas) and galactooligosaccharides are selectively fermented by these microbes, boosting their growth and increasing SCFA production. Prebiotic supplementation has been shown to strengthen the intestinal mucus layer, reduce pro-inflammatory cytokines, and improve microbiota composition in experimental colitis. For example, oral inulin reduced disease severity in mouse colitis models by inhibiting IL-6/STAT3 signaling and elevating mucin (MUC2) levels. Such findings suggest that prebiotics actively contribute to intestinal homeostasis and immune regulation, acting as a proactive defence against dysbiosis that can precede IBD. Indeed, a recent review highlights that prebiotic fibre interventions modulate gut immunity—for instance, inulin increased anti-inflammatory Th2 responses and lowered pro-inflammatory cytokines in preclinical studies [112].

#### 6.1.3. Omega-3 Polyunsaturated Fatty Acids (PUFAs)

Diets rich in omega-3 PUFAs (particularly eicosapentaenoic acid, *EPA,* and docosahexaenoic acid, *DHA*, found in fatty fish) appear to protect against IBD development. These fatty acids are precursors to specialised pro-resolving mediators that actively resolve inflammation rather than simply suppress it. Epidemiological studies and genetic analyses have observed that higher omega-3 intake (or circulating levels) is associated with a significantly decreased risk of both Crohn’s disease and ulcerative colitis. In contrast, high omega-6 intake (e.g., linoleic acid) has been linked to increased risk of UC. A recent Mendelian randomisation study confirmed a causal protective effect of omega-3 (especially DHA) on IBD: individuals genetically predisposed to higher DHA had ~19% lower odds of IBD. By shifting the balance of eicosanoids toward less pro-inflammatory and more pro-resolving mediators, a diet enriched in omega-3 fatty acids may mitigate early immune triggers for IBD [113].

#### 6.1.4. Limiting Processed Foods and Additives

Conversely, Western-style diets high in ultra-processed foods (UPFs) are associated with heightened IBD risk. Processed foods often contain emulsifiers, artificial sweeteners, and high saturated fat, which can adversely affect the gut microbiome and barrier. A recent prospective study of over 240,000 adults found that those in the highest quartile of UPF consumption had a 70% higher risk of developing Crohn’s disease compared to those in the lowest quartile [92]. Mechanistic research offers insight: emulsifier additives (like polysorbate-80 and carboxymethylcellulose) can erode the mucus layer and promote bacterial encroachment, triggering colonic inflammation. Likewise, certain artificial sweeteners and a high-sugar/high-fat diet can favour pro-inflammatory microbial species and increase intestinal permeability. Reducing these dietary components—especially in early life—may thus lower IBD susceptibility. Notably, early-life exposure to unhealthy diets during critical immune development windows is thought to “program” a more inflammation-prone microbiota–immune setpoint. In a pooled analysis of Scandinavian birth cohorts (over 80,000 children), a high-quality diet in infancy (characterised by greater fish and vegetable intake and less sugary beverages) was associated with a significantly reduced risk of IBD later in childhood and adolescence. For example, children at 1 year old with high fish intake had about a 30–50% lower risk of IBD (particularly ulcerative colitis) compared to those with low fish intake. These findings underscore that fostering healthy eating patterns from infancy—and minimising early exposure to Western diet elements—can build a more resilient flora–immune axis.

### 6.2. Secondary Treatment

#### 6.2.1. Fecal Microbiota Transplantation

Fecal microbiota transplantation (FMT), as a method of directly re-establishing intestinal flora, aims to restore the intestinal microbial ecology of IBD patients by transplanting faeces from healthy donors. However, while FMT has shown high remission rates in UC, its efficacy in CD remains controversial [85]. Next-generation FMT technology should require SCFA producer abundance > 40% for donor screening to ensure the quality and functionality of the transplanted flora. Engineered bacterial design is an emerging technology for ecological remodelling of flora in IBD patients; e.g., Nissle 1917, an engineered *E. coli* that secretes IL-2, modulates immune response and improves intestinal inflammation [114].

#### 6.2.2. Probiotics

Probiotics, live microorganisms conferring health benefits when administered in adequate amounts, represent a targeted approach to microbial modulation [115]. Regarding immune memory, certain probiotic strains bidirectionally influence trained immunity. *Lactiplantibacillus plantarum* induces tolerogenic trained immunity by reprogramming mononuclear phagocytes to reduce pro-inflammatory cytokine release upon restimulation, promoting immune homeostasis [50]. However, conventional probiotics demonstrate limited therapeutic efficacy in IBD due to poor gastrointestinal survival and transient, inefficient mucosal colonization [115,116]. To overcome the limitations of conventional probiotics, research has shifted towards “next-generation” engineered probiotics, a frontier of synthetic biology. These “smart” microbes are rationally designed to function as living medicines, capable of sensing environmental cues and delivering therapeutic payloads with high precision directly at the site of inflammation. A prominent strategy involves engineering safe bacterial chassis, such as *E. coli* Nissle 1917, to produce and secrete specific immunomodulatory molecules. For example, strains have been developed to release Interleukin-10 (IL-10) or other anti-inflammatory cytokines [117], which can locally suppress aberrant immune responses and promote mucosal healing. More advanced designs incorporate sensor–actuator circuits, enabling bacteria to detect specific inflammatory markers in the gut, such as tetrathionate or nitric oxide [118], and trigger the production of a therapeutic agent only when and where it is needed. This “sense and respond” approach minimises systemic exposure and off-target effects, representing a significant leap towards personalized and dynamic IBD treatment.

#### 6.2.3. Postbiotics

The challenges associated with live probiotics have led to a growing interest in postbiotics, defined as a “preparation of inanimate microorganisms and/or their components that confers a health benefit on the host”. As a therapeutic strategy for IBD, postbiotics offer significant advantages over live microbes, including enhanced stability, a superior safety profile, and the capacity for precise dose standardisation. Clinical evidence supporting the efficacy of postbiotics in IBD, while still emerging, is growing. The most robust data comes from trials of topical butyrate administration in UC. In a multicenter trial, Vernia et al. demonstrated that the combination of butyrate and 5-ASA enemas significantly improved clinical parameters such as bowel movements and urgency compared to 5-ASA alone, suggesting a potential benefit in refractory distal UC [119]. Furthermore, studies using heat-inactivated probiotics have demonstrated potential. In an acute colitis mouse model, heat-inactivated *Lactiplantibacillus plantarum* L-137 treatment improved intestinal barrier integrity by upregulating ZO-1 and modulating cytokine levels, leading to an anti-inflammatory effect [120]; similar benefits are reported for inactivated lactobacilli and bacterial lysates that reduce pro-inflammatory cytokines and increase IL-10 [121]. Complementing these findings, oral administration of a composite postbiotic derived from *Lacticaseibacillus casei* Zhang, *Lactiplantibacillus plantarum* P-8, and *Bifidobacterium animalis* subsp. lactis V9 ameliorated DSS-induced colitis in rats in a dose-dependent manner, lowering TNF-α and IL-1β, increasing IL-10, and shifting the microbiota and fecal metabolome toward anti-inflammatory profiles [122].

Additionally, metabolic and epigenetically targeted therapies offer new avenues for the treatment of IBD chronicity. The innate immune cell-expressed G protein-coupled receptor GPR84 is significantly increased in response to acute inflammatory stimuli such as LPS and TNFα, and its antagonists inhibit colitis by decreasing the polarisation and function of pro-inflammatory microphages [123]. AhR agonists enhance polyamine biosynthesis, modulate immune responses, and maintain intestinal immune homeostasis [124].

### 6.3. Tertiary Management

In the long-term management stage, the dietary management of IBD patients is crucial. Previous studies have concluded that environmental factors (e.g., high-fat, high-sugar WD, and antibiotic use), rather than genetic factors, drive changes in flora structure and exacerbate intestinal permeability and inflammation [86,125,126]. However, some animal studies have shown that brief WD intake leads to beneficial immune training via the mevalonate pathway and macrophage training, which is protective against DSS-induced colitis [127]. In addition, modulation of the intestinal biological clock through diet is beneficial in ameliorating persistent inflammation due to dysbiosis [95].

Periodisation of treatment is important to prevent the recurrence of IBD. In addition, dynamic detection of flora-related markers is important. By monitoring the dynamic changes in clinical markers, we can understand the progress of patients’ conditions in real time, adjust the treatment strategy in time, improve the therapeutic effect, and improve patients’ prognosis.

## 7. Challenges and Future Directions

Despite significant advances in understanding the gut microbiota–immunity axis, key challenges persist across clinical practice, therapeutic translation, and fundamental science. Clinically, the disconnect between patient-reported symptoms and objective inflammatory activity, combined with limitations of current assessment tools, complicates therapeutic decision-making and evaluation of novel interventions [128,129,130]. These challenges are magnified when translating microbiome-based therapies from laboratory to clinic.

Future research must integrate multi-omics data to elucidate precise interactions between host genetics, microbiome, and immune memory [131]. Personalized IBD therapy requires understanding how genetic variants shape both microbiome composition and immune responses to specific therapies. For instance, patients with certain risk alleles may exhibit unique microbial metabolic signatures that, in turn, influence the state of trained immunity and the efficacy of a given treatment [132]. Integrating genotype, microbiome profile, and immune phenotype enables development of predictive biomarkers for treatment response and disease progression, facilitating true precision medicine through a “genetics–microbiota–immunity” axis approach.

For FMT, major clinical obstacles include unstandardised donor screening and administration protocols, leading to variable outcomes and unpredictable engraftment, alongside persistent long-term safety concerns over pathogen transmission [133,134,135,136]. Next-generation Live Biotherapeutic Products face manufacturing challenges in ensuring batch consistency and stability, compounded by regulatory complexities due to ambiguous classification and demanding Chemistry, Manufacturing, and Controls requirements that slow clinical translation.

While current postbiotic evidence in IBD is promising, larger randomised controlled trials are essential to confirm efficacy and establish standardised protocols. Future research should optimise formulations, determine dosing regimens, and identify responsive patient subgroups. The demonstrated mechanisms—mitochondrial modulation, microbiome–metabolome reprogramming, and targeted anti-inflammatory effects [121,122]—position postbiotics as precision therapeutics with superior safety and stability profiles. As mechanistic understanding and clinical evidence expand, postbiotics offer significant promise for transforming IBD treatment and achieving sustained remission.

Scientifically, causal relationships between dysbiosis and IBD remain incompletely defined [137]. More specifically, direct links between microbial community shifts and the balance of anti- versus pro-inflammatory microbial metabolites are not yet resolved; for example, IBD has been associated with increased succinate-producing taxa and with excessive polyamine levels linked to Bacteroides enrichment [138,139,140,141]. Although there is suggestive evidence that pro-inflammatory metabolites such as succinate can modulate trained immunity in inflammatory settings, robust experimental and clinical studies confirming causality remain limited in IBD [142]. Additionally, certain non-immune cell types, such as epithelial cells, endothelial cells, smooth muscle cells, and fibroblasts, can also establish inflammatory memory in a manner similar to monocytes and macrophages [23]. The definition of trained immunity may be further expanded, and whether inflammatory memory in non-immune cells exists in IBD warrants further exploration.

Addressing these challenges requires interdisciplinary innovation integrating personalized multi-omics analysis, real-time mucosal metabolic imaging, and digital twin models to dissect patient-specific mechanisms. Next-generation precision interventions show promise, including synthetic biology-modified probiotics engineered for inflammation sensing, organoid-based immune models for personalized therapy screening, and nanocarrier-mediated therapeutic delivery [63,143]. Furthermore, microbiota-targeted vaccines and gene editing technologies represent emerging frontiers, though their efficacy, safety, and translational pathways require validation [70,144,145,146]. Overcoming these barriers is essential for developing effective, personalized IBD therapeutics.

## 8. Conclusions

The traditional conceptualization of inflammatory bowel disease (IBD) as a simple dysregulation between pro- and anti-inflammatory mediators is undergoing fundamental revision. Our comprehensive review elucidates a more sophisticated paradigm, wherein maladaptive trained immunity, orchestrated and sustained by gut microbiome dysbiosis, constitutes a central pathogenic axis driving IBD chronicity. This “gut microbiome–immune memory” circuit provides mechanistic insight into the persistent, relapsing nature of IBD and, critically, unveils numerous novel therapeutic targets.

The future management of IBD is positioned to transition from broad-spectrum immunosuppression toward an era of precision, mechanism-based therapeutics. The targeted recalibration of maladaptive trained immunity—through strategic deployment of postbiotics to restore metabolic and epigenetic homeostasis, rationally engineered probiotics functioning as “living medicines”, or genetically informed nutritional interventions—represents the next therapeutic frontier in IBD management. By addressing the fundamental pathophysiological drivers rather than merely suppressing downstream inflammatory cascades, these precision strategies offer the potential not only for symptomatic control but for the restoration of mucosal homeostasis and achievement of sustained, treatment-free remission.

This paradigm shift from reactive immunosuppression to proactive immune reprogramming heralds a transformative approach to IBD therapeutics, with the ultimate goal of disease modification rather than mere disease management.

## Figures and Tables

**Table 1:** Similarities and differences between trained immunity and adaptive immunity in IBD pathogenesis.

	Trained Immunity	Adaptive Immunity
Basic features		
Major cell types	monocytes/macrophages; NK cells; (HSPCs) [23]	T cells [24]
Response specificity	Non-specific; broad cross-protection	Highly antigen-specific
Anatomical localization	Bone marrow hematopoietic niche plus tissue-resident compartments [23]	Lymphoid organs plus circulation
Molecular mechanisms		
Basis of memory formation	Epigenetic reprogramming plus metabolic remodelling [23]	Gene rearrangement plus clonal expansion [25]
Key cellular alterations	Macrophage polarization imbalance—M1 (pro-inflammatory)/M2 (anti-inflammatory); pro-inflammatory bias contributes to IBD [26] Monocyte subset imbalance—MCI (enhanced inflammatory)/MC (anti-inflammatory) [11,27]	Th/Treg imbalance promotes intestinal inflammation [28] Innate immune abnormalities perturb adaptive immune balance [29]
Key signaling pathways	TLR signaling [30] mTOR/HIF-1α metabolic regulation [11,31] AhR signaling [32]	cytokines/JAK–STAT [33,34] co-stimulatory signal imbalance [35]
Functional features		
Memory maintenance (partial mechanisms)	Persistent epigenetic reprogramming of bone marrow HSPCs [16] in situ training of tissue-resident cells [23] metabolic memory sustaining activation [26] gut–bone marrow axis remote regulation [36]	Long-term survival and reactivation of T-cell subsets [28]
Cross-reactivity	Broad recognition of PAMPs/DAMPs; cross-reactivity across pathogens; tolerance induction [30]	Precise epitope recognition; antigen-specific anergy or deletion [37]
Commonality	Trained and adaptive immunity are interlinked, with bidirectional crosstalk shaping IBD pathogenesis [25,38]	

**Table 2:** Microbial agents, components, and metabolites that induce or modulate innate immune memory (trained immunity or tolerance) in experimental models.

Microbial Agent/Component	Target Cell/Model	Mechanism/Effect	Key Notes	Ref.
**Commensal bacteria (strain-specific)**				
***Saccharomyces cerevisiae*, *L. plantarum***	**Human monocytes**	**Single strain induces trained immunity (↑TNF-** **α, ↑IL-6); combined stimulation induces tolerance (↑IL-10, ↓CD14/CD86).**	**Effect depends on strain, dose, and co-stimulation.**	[49]
***L. plantarum***	**Murine/human monocytes/macrophages**	**Induces anti-inflammatory memory phenotype (↑IL-10, ↓pro-inflammatory cytokines, ↓ROS); enhances bacterial survival.**	**Requires live bacteria; dose-dependent effect.**	[50]
***Akkermansia muciniphila***	**Murine/human macrophages, monocytes**	**Induces trained immunity phenotype (↑bacterial survival, ↓TNF/IL-10), metabolic rewiring (↑glycolysis, ↓lysosome), ↑tolerogenic response.**	**Requires live bacteria; not replicated with the heat-killed form.**	[51]
***Limosilactobacillus reuteri***	**Human dendritic cells (mo-DCs)**	**Atypical memory: ↑IL-6/IL-1** **β, ↓TNF** **α/IL-23/IL-27; promotes Th17/Th9 differentiation.**	**Effect attenuated by retinoic acid; secreted bacterial factors.**	[52]
**Commensals—Tissue/Immune niche effects**				
**Enteric commensals, LRCs, etc.**	**Murine mucosal DCs & ILCs**	**DCs retain commensals, induce IgA, local cytokines (IL-10, IL-22); foster tolerance and tissue protection.**	**Site-restricted; live bacteria; IL-10 and TLR-dependent regulation.**	[54,55]
**Pathogen exposure/vaccine**				
***Citrobacter rodentium* (infection)**	**Murine gut ILC3**	**Generates long-lived trained ILC3s (↑IL-22, ↑proliferation, better control); stable transcriptional/metabolic reprogramming.**	**Non-specific, transferable, adaptive immunity-independent.**	[53]
**BCG vaccine**	**Human monocytes**	**Epigenetic reprogramming (↑H3K27ac/H3K4me3), ↑IL-1** **β/TNF** **α/IL-6; heterologous protection.**	**IL-1β pathway polymorphisms influence response.**	[40]
**Influenza vaccine (inactivated)**	**Human PBMCs, clinical cohort**	**Induces trained immunity (monocyte reprogramming, ↑cytokines), lowers systemic inflammation.**	**Associated with reduced risk of COVID-19 in epidemiological studies.**	[61]
**Microbial components/metabolites**				
**β-glucan (*Candida albicans*, others)**	**Murine/human monocytes**	**Enhanced TNFα/IL-6 (trained immunity), Dectin-1/Raf-1/H3K4me3-dependent; protection in T/B-deficient mice.**	**Dose/context-dependent; antifungal activity; long-lasting effect.**	[60]
**Bacterial LPS**	**Gut epithelium, macrophages, T cells**	**Via TLR4→NF-** **κB: ↑pro-inflammatory cytokines, M1 polarization, barrier disruption, Th1/Th17 skewing, chronic inflammation.**	**LPS effect varies by species, dietary context, and gut environment.**	[58]
**LPS/HIF-1α/mTORC1 axis**	**Mouse/human macrophages**	**Epigenetic (↑H3K4me3) and metabolic (↑glycolysis, HIF-1** **α/mTORC1) reprogramming; persistent pro-inflammatory trained phenotype.**	**Glycolysis and HIF-1α are required for sustained output.**	[59]
**Short-chain fatty acids (butyrate, etc.)**	**Human PBMCs, murine macrophages**	**Induce tolerogenic/trained memory phenotypes, modulate cytokine output, alter chromatin state, and enhance pathogen killing.**	**Effect is context- and differentiation-dependent.**	[12]
**Polyvalent**				
**MV130 (poly-bacterial vaccine)**	**Mouse BM progenitors, human monocytes**	**Epigenetic/metabolic reprogramming; ↑recall cytokines; broad protection from viral/fungal infection; mTOR-dependent.**	**Metformin blocks the effect; protection lasts months.**	[62]

## Abbreviations

The following abbreviations are used in this manuscript:

IBD	Inflammatory bowel disease
CD	Crohn’s disease
UC	Ulcerative colitis
TCA	Tricarboxylic acid cycle
NOD2	Nucleotide-binding oligomerisation domain-containing protein 2
CARD9	Caspase recruitment domain-containing protein 9
NK	Natural killer
lncRNAs	Long non-coding RNAs
HIF-1α	Hypoxia-inducible factor 1-alpha
mTOR	Mechanistic target of rapamycin
HSPCs	Hematopoietic stem and progenitor cells
SSc	Systemic sclerosis
HDAC	Histone deacetylase
SCFA	Short-chain fatty acid
AhR	Aryl hydrocarbon receptor
CNS	Central nervous system
γδ T	Gamma delta T cells
Th17	T helper 17
GABA	Gamma-aminobutyric acid
APOL9a/b	Apolipoprotein L9a/b
OMVs	Outer membrane vesicles
TLR	Toll-like receptor
IFN-γ	Interferon gamma
MHC-II	Major histocompatibility complex class II
NF-κB	Nuclear factor kappa B
MCI	Monocytes with enhanced inflammatory properties
MC	Monocytes with anti-inflammatory properties
GWASs	Genome-wide association studies
DSS	Dextran sulfate sodium
Mincle	Macrophage-inducible C-type lectin (CLEC4E)
ZO-1	Zonula occludens-1
LPS	Lipopolysaccharide
CX3CR1	C-X3-C motif chemokine receptor 1
STING	Stimulator of interferon genes
TBK1	TANK-binding kinase 1
IRF3	Interferon regulatory factor 3
GMPs	Granulocyte–monocyte progenitors
WD	Western diet
FMT	Fecal microbiota transplantation
IL-2	Interleukin-2
EPA	Eicosapentaenoic acid
DHA	Docosahexaenoic acid
UPFs	Ultra-processed foods
STAT3	Signal transducer and activator of transcription 3
MUC2	Mucin 2
Th	T helper cell
Treg	Regulatory T cell
PAMPs	Pathogen-associated molecular patterns
DAMPs	Damage-associated molecular patterns
APC	Antigen-presenting cell
